# Anti-C1q Autoantibody-Binding Engineered scFv C1q-Mimicking Fragment Enhances Disease Progression in Lupus-Prone MRL/lpr Mice

**DOI:** 10.3390/ijms26157048

**Published:** 2025-07-22

**Authors:** Silviya Bradyanova, Nikolina Mihaylova, Nikola Ralchev, Alexandra Kapogianni, Ginka Cholakova, Kalina Nikolova-Ganeva, Ivanka Tsacheva, Andrey Tchorbanov

**Affiliations:** 1 Department of Immunology, Institute of Microbiology, Bulgarian Academy of Sciences, 1113 Sofia, Bulgaria; silvybradyanova@microbio.bas.bg (S.B.); mihaylova_n@microbio.bas.bg (N.M.); nikola_ralchev@microbio.bas.bg (N.R.); nikolova_k@microbio.bas.bg (K.N.-G.); 2 Department of Biochemistry, Faculty of Biology, Sofia University “St. Kliment Ohridski”, 1000 Sofia, Bulgaria; kapojanni@uni-sofia.bg (A.K.); ginka.cholakova@biofac.uni-sofia.bg (G.C.); itsacheva@biofac.uni-sofia.bg (I.T.)

**Keywords:** systemic lupus erythematosus, mouse models, anti-C1q autoantibodies

## Abstract

Systemic lupus erythematosus (SLE) is a chronic inflammatory autoimmune disease characterized by tissue damage in multiple organs caused by autoantibodies and the resulting immune complexes. One possible way for complement system contribution to onset of autoimmune disorder could be realized by the impairment of C1q-mediated apoptotic clearance as part of human homeostasis. The capacity of C1q to bind early apoptotic cells could be decreased or even lost in the presence of anti-C1q antibodies. A monoclonal anti-idiotypic single-chain (scFv) antibody was selected from the phage library Griffin1” to recognize anti-C1q autoantibodies, purified from sera of lupus nephritis patients. Lupus-prone MRL/lpr mice were injected weekly with scFv A1 fragment-binding anti-C1q antibodies. The number of in vitro and ex vivo studies with collected cells, sera, and organs from the treated animals was performed. scFv treatment changed the percentage of different B-, T-, and NK-cell subpopulations as well as plasma cells and plasmablasts in the spleen and bone marrow. An increase in the levels of splenocyte proliferation, anti-C1q antibodies, and the number of plasma cells producing anti-dsDNA and anti-C1q antibodies were also observed in scFv-treated animals. High levels of proteinuria and hematuria combined with unstable levels of IL10 and IFNγ promote the development of severe lupus and shorten the survival of treated MRL/lpr mice. Therapy with the scFv A1 antibody resulted in BCR recognition on the surface of anti-C1q-specific B-cells and had a disease progression effect, enhancing lupus symptoms in the MRL/lpr mouse model of SLE.

## 1. Introduction

Systemic lupus erythematosus (SLE) is a severe systemic autoimmune disease with multiple organ involvment and disregulated self-tolerance. Genetic predisposition, sex hormones, epigenetic, and environmental factors are implicated in the initiation of the pathological processes followed by B- and T-cell hyperactivity and generation of a diverse array of disease-associated auto-antibodies [1,2,3]. Although specific and effective treatment for SLE is still lacking, it relies on immunosuppression by cyclophosphamide and glucocorticosteroids, as well as by monoclonal antibodies [4,5]. The pathological cooperation between the pair of activated self-reactive B- and T-lymphocytes is a main factor for SLE development, although both cell subsets may have also independent functions for disease progression [6,7]. For instance, pro-inflammatory cytokines secretion from self-specific T-cells amplify lupus pathogenesis by regulation of B-cell activity and autoantibody production, or via several B-cell-independent pathways. In opposite, in addition to the autoantibody secretion, autoreactive B-lymphocytes play as antigen-presenting cells inducing the loss of T-cell tolerance and modifying the cytokine production [8,9,10,11].

Despite the widely used classical immunosuppressants, the B-cell-targeted therapies are based on application of monoclonal antibodies against pan-B-cell surface markers expressed on all mature B-cells in the periphery. The general B-cell depletion by engaging such molecules as CD19, CD20 (rituximab, ocrelizumab), and CD22 (epratuzumab) has successful clinical introduction and partial benefit after administration for several autoimmune diseases [5,8,12,13,14,15]. However, this approach remains unspecific, risky, and inefficient in many clinical cases, requiring new design of therapy for selective elimination of autoreactive B-cell populations only.

The complement system deficiency is a determinative factor for lupus development and primary deficiencies of the early components of the classical pathway (C1q, C4, C2) are recognized as the strongest genetic predisposition for SLE. Acquired C1q deficiency is directly linked to development and severity of SLE. While the primary C1q deficiency is not frequent among SLE patients, 20–50% of them develop elevated levels of anti-C1q autoantibodies, which may be responsible for low C1q levels. The importance of these antibodies is due to the observed correlation between their occurrence and the severity of lupus nephritis. Anti-C1q antibodies are considered participants in the pathogenesis of SLE and their elimination is a reasonable approach for disease control [16,17,18].

C1q has a complex biochemical structure of 18 polypeptide chains of three types, namely A, B, and C, which form two functional types of domains—a collagen-like (CLR) domain at the N-terminus and six globular domains (gC1q) at the C-terminus. Each gC1q consists of the globular fragments of the three types of chains, designated as ghA, ghB, and ghC. The latter show structural and functional autonomy underlying the versatile ligand binding of C1q [19].

A monoclonal single-chain (scFv) antibody, selected from the phage library Griffin.1 provides a source of anti-idiotypic scFv specific for anti-C1q autoantibodies purified from the sera of lupus nephritis patients. These recombinant monovalent antibodies have been shown to be inhibitors of the C1q–anti-C1q antibody interaction under in vitro conditions [20]. The intriguing question was how this engineered molecule would affect the complex immune system during in vivo therapy of lupus mice.

The murine models of SLE represent different features of human disease, being important tool for new drugs investigation. Among them, MRL/lpr mice develop spontaneously a wide panel of autoantibodies and a variety of autoimmune symptoms closely overlapping the human lupus [6,7]. Previously, we employed an MRL/lpr mouse model in order to suppress disease activity by treatment with bi-functional chimeric antibody which cross-links the B-cell receptor and the inhibitory FcγRIIb receptor on the surface of anti-dsDNA antibody-producing lymphocytes as well as by a monoclonal anti-ANX A1 antibody [21,22,23]. In the present research, we evaluated the effect of long-term therapy with scFv specific for anti-C1q autoantibodies in MRL/lpr mice on the development of murine systemic lupus erythematosus.

## 2. Results

### 2.1. scFv A1 Production

**Figure 1:**
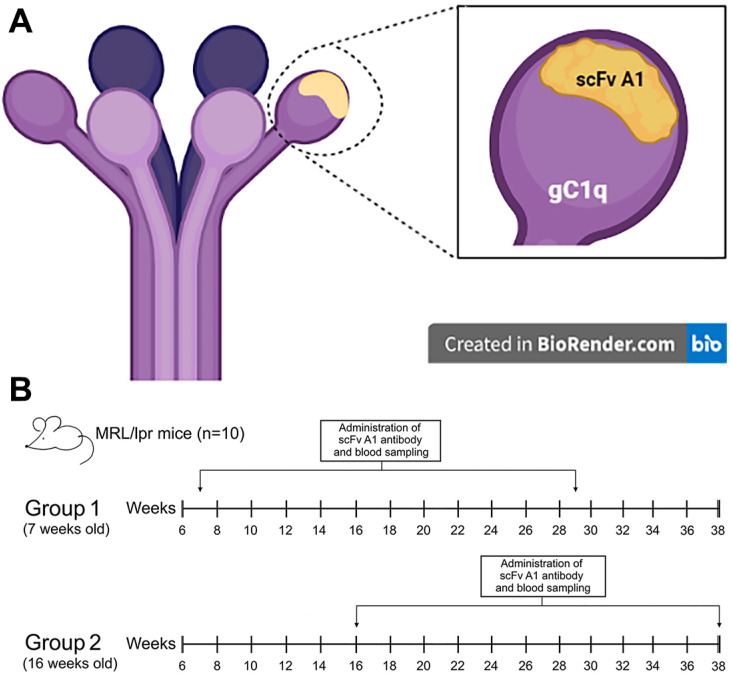
(**A**) Graphical representation of C1q structure and positional overlap of scFv A1 antibody; (**B**) the therapeutic design of in vivo experiments. Created in BioRender. Cholakova G. (2021). https://app.biorender.com/illustrations/60f7fd96bfd89a00a50f2afb.

The synthesis of the soluble form of the monoclonal scFv A1 was induced in the non-suppressor strain of *E. coli* HB 2151. The recombinant antibody was selected as an anti-idiotype of anti-C1q antibodies, affinity purified from human sera of lupus nephritis patients, and was found to inhibit the recognition of C1q as well as its globular domains (gC1q) by the lupus autoantibodies. Each gC1q is composed of three globular fragments, namely ghA, ghB, and ghC, representing the C-terminal halves of the three chain types A, B, and C, respectively, which comprise the protein C1q. The anti-idiotype scFv A1 is known to be a structural analogue of the apical area of gC1q, including portions of all three globular fragments, thus localizing a globular autoepitope of C1q (Figure 1A).

### 2.2. Treatment of MRL/lpr Mice with a scFv A1

The scheme of treatment of experimental mice with a scFv A1 antibody is shown in Figure 1B.

### 2.3. Phenotyping of Splenocytes and Bone Marrow Cells

Quantitative FACS analysis of the cells isolated from spleens and bone marrows was performed for treated and control mice to evaluate the effect of scFv A1 administration. Numerous differences in the lymphocytes’ quantity were found following the scFv A1 antibody treatment either in both 7- and 16-week-old MRL/lpr mice, compared to the untreated controls.

**Figure 2:**
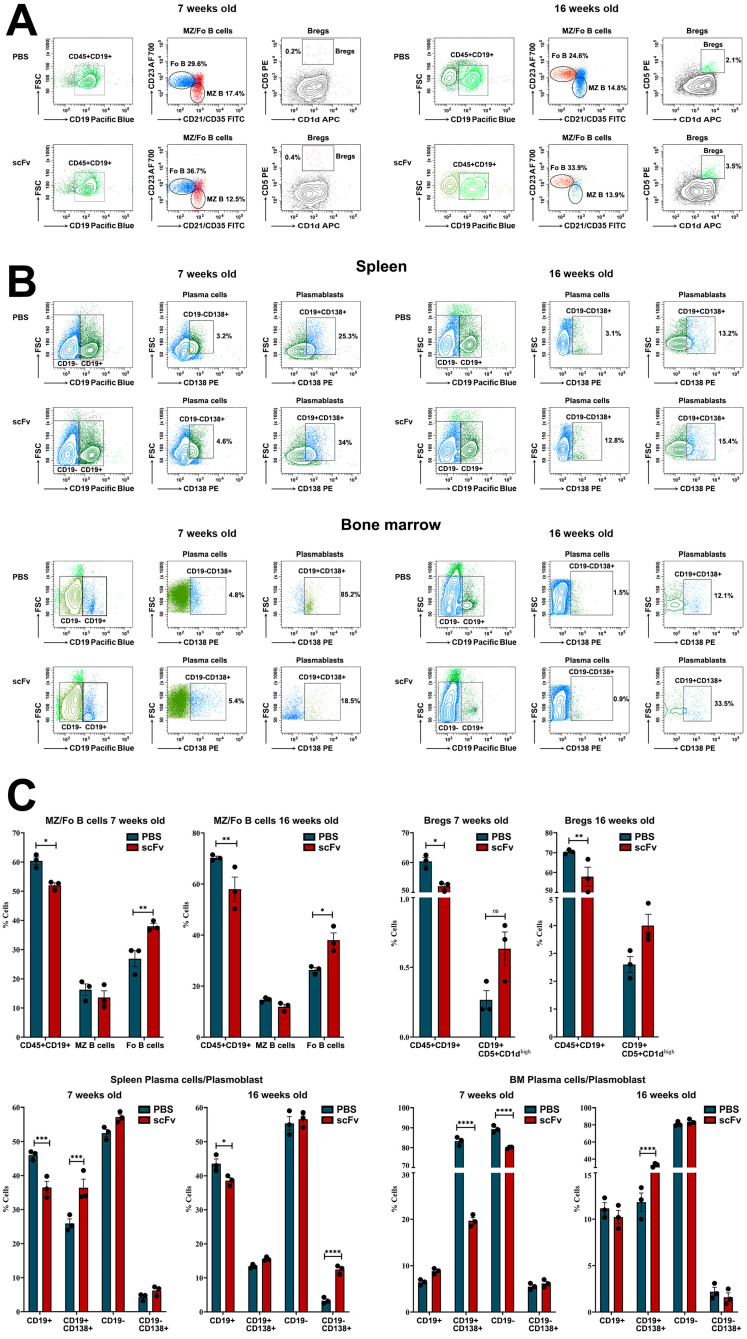
Phenotyping of B-cells, plasma cells, and plasmablasts in spleens and bone marrow of MRL/lpr mice from all groups. Cell suspensions were prepared as described above and incubated with one of the anti-mouse antibody mixes described in the Materials and Methods section. Thirty thousand lymphocyte-gated cells from each tube were collected and analyzed by flow cytometry. Representative data from 3 experiments are shown for analysis of Bregs and Mz/Fo B-cells in the spleen (**A**) and plasma cells and plasmablasts in the spleen and bone marrow (**B**). The extracted results obtained from all experiments are presented graphically as a percentage of total viable immune cells (**C**). The data are represented as mean ± SD, *p* values were calculated using the two-way ANOVA test (*n* = 3, * *p* < 0.05; ** *p* < 0.01; *** *p* < 0.001; **** *p* < 0.0001) compared to values from control mice.

An increased number of Follicular B-cells (Fo B, CD45R+CD19+CD1d−CD23+) and regulatory B-cells (Bregs, CD19+CD5+CD1d+) were observed in the spleens of both groups (young and old mice) treated with scFv A1 antibody compared to PBS injected animals. In contrast, a decrease in the percentage of Marginal zone B-lymphocytes (MZ B, CD45R+CD19+CD1d+CD21/CD35+) was found in both scFv A1 antibody-treated groups after the therapy (Figure 2A,C).

Further, we followed the plasma cells (CD19−CD138+)/plasmablasts (CD19+CD138+) ratio in spleen and bone marrow (Figure 2B,C). Analyzing the splenocytes, a significant increase was found in the percentage of both plasma cells and plasmablasts into the scFv A1 antibody-treated young and old animals compared to controls. The lymphocytes isolated from bone marrow also exhibited an increase in the plasma cell number in young mice and of plasmablasts in old mice after scFv A1 antibody therapy. An opposite tendency was monitored for plasmablasts in young mice and for plasma cells in old mice treated with scFv A1 (Figure 2B,C).

**Figure 3:**
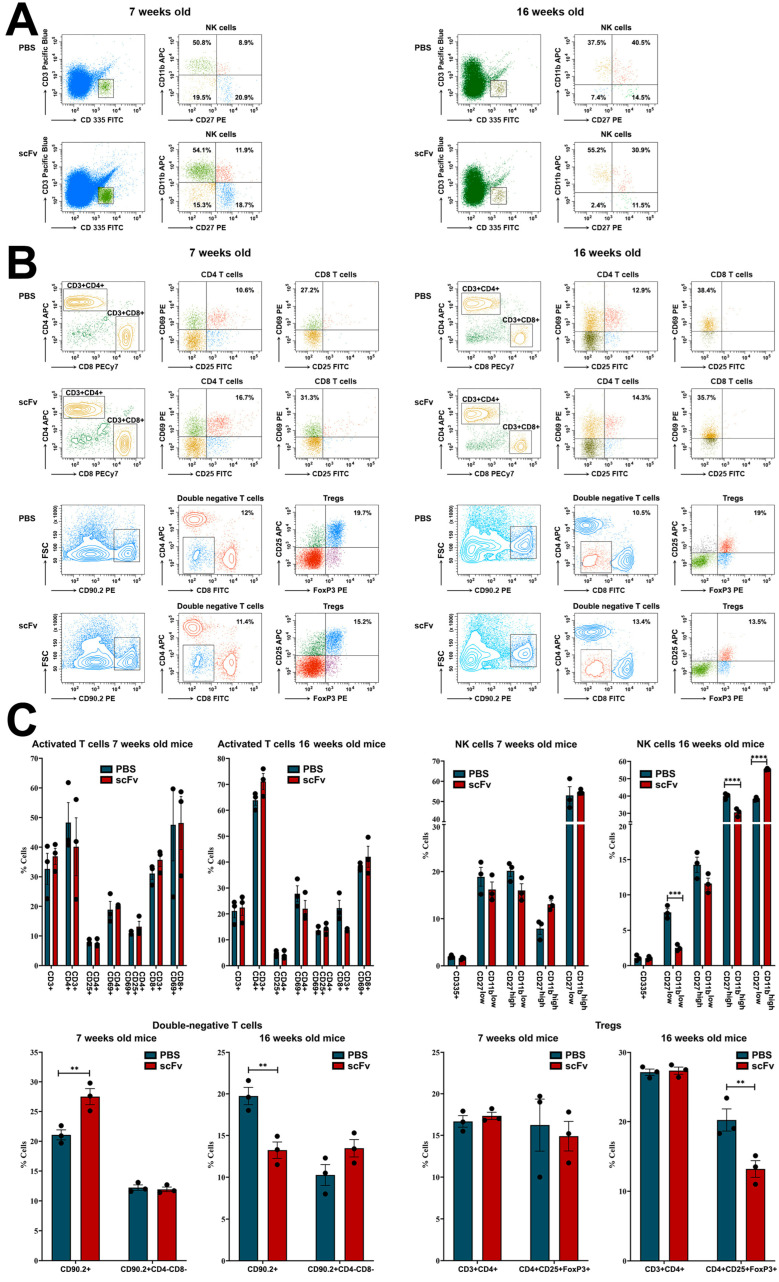
Phenotyping of NK- and T-cells in spleens of MRL/lpr mice from all groups. Cell suspensions were prepared as described above and incubated with one of the anti-mouse antibody mixes described in the Materials and Methods section. Thirty thousand lymphocyte-gated cells from each tube were collected and analyzed by flow cytometry. Representative data from 3 experiments are shown for analysis of NK-cells (**A**) and T-cells (**B**). The extracted results obtained from all experiments are presented graphically as a percentage of total viable immune cells (**C**). The data are represented as mean ± SD, *p* values were calculated using the two-way ANOVA test (*n* = 3, ** *p* < 0.01; *** *p* < 0.001; **** *p* < 0.0001) compared to values from control mice.

Moving back to splenocytes, lower levels of NK-cells in early maturation stage (CD27+CD11b−) were monitored in both young and old groups of mice treated with scFv A1 antibody relative to control animals, while higher levels of cytotoxic NK-cells (CD27−CD11b+) were found also in both young and old sick mice administered with scFv A1. Age-related differences were found in the mature (CD27+CD11b+) NK-cells: an increased number in scFv A1-treated young animals, and decreased number in the old mice after the same therapy (Figure 3A,C).

T-cell populations in the spleen exhibit specific features in lupus-prone MRL/lpr mice. Activated (CD25+CD69+) CD4+ T-cell subpopulation showed a huge content of this subset in both young and old animals treated with scFv A1, but an increase in activated (CD69+) CD8+ T-cells was found only in young mice after the therapy. The 16-week-old sick MRL/lpr mice treated with single-chain antibody had lower levels of activated CD8+ T-lymphocytes compared to controls (Figure 3B,C).

A decrease in CD25+FoxP3+ regulatory T-cells (Tregs) was observed in both young and old mice after scFv A1 therapy, while a weak decrease in double negative CD4−CD8− cells was found in young animals only. The increased number of CD4−CD8− T-lymphocytes was monitored in the scFv A1-treated group of sick old MRL/lpr mice (Figure 3B,C).

### 2.4. scFv A1antibodies Stimulate the Cell Proliferation and the Generation of Anti-dsDNA and Anti-C1q Antibody-Secreting Cells in Vitro

**Figure 4:**
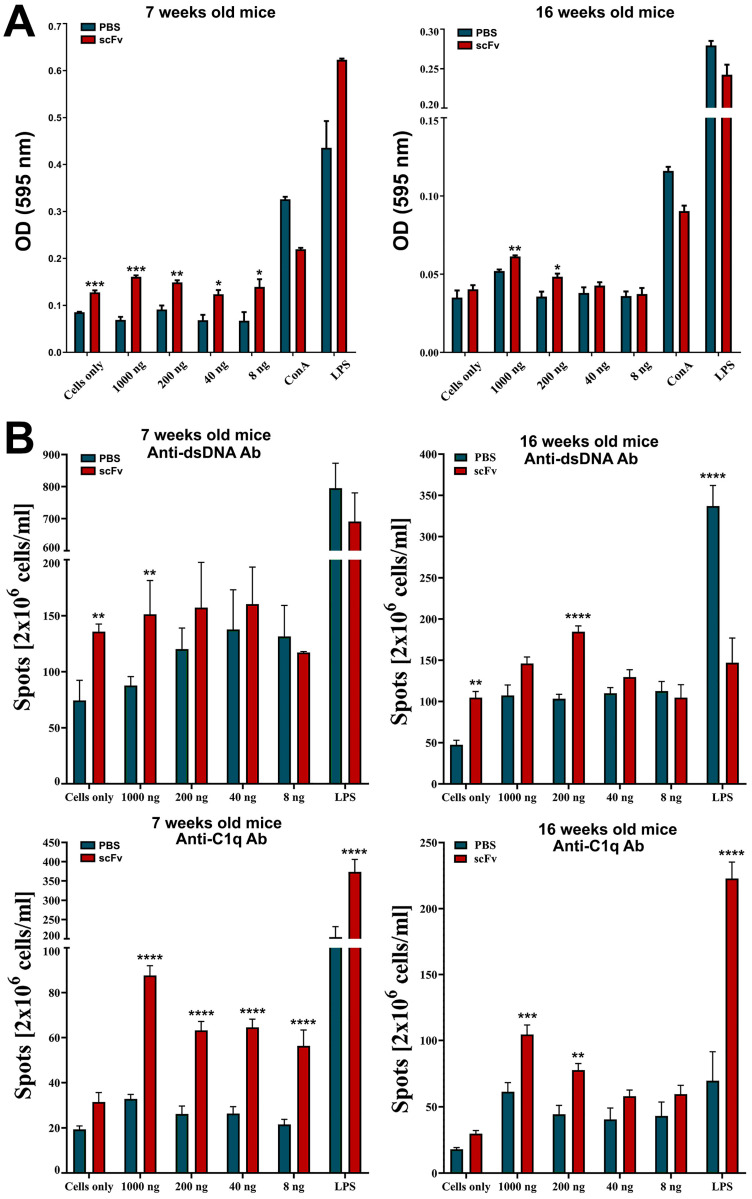
Treatment of MRL/lpr splenocytes with scFv A1 antibody enhanced cell proliferation and increased the number of anti-dsDNA and anti-C1q antibody-secreting cells ex vivo. Spleen cells from all MRL/lpr mice were cultured in the presence of various concentrations of scFv A1 antibody. Control splenocytes were stimulated with ConA, or with LPS, or cultured in medium alone. Samples from in vivo scFv A1 antibody-treated MRL/lpr mice were compared with cells from untreated animals. (**A**) The scFv A1 antibody stimulated cell proliferation ex vivo in both young and old groups of animals. (**B**) Treatment with scFv A1 antibody ex vivo increased the number of cells secreting anti-dsDNA and anti-C1q antibodies in both young and old mice in groups treated in vivo with the same engineered molecule. The number of plasma cells secreting anti-dsDNA and anti-C1q-specific IgG antibodies was determined using an ELISpot assay. Spot counts in test wells from in vivo treated animals were compared with wells containing splenocytes from control mice. All samples were triplicated and the average values were used for analysis. Results are expressed as the mean value ± SD of triplicated assays; *p* values were calculated using a two-way ANOVA test (*n* = 5, * *p* < 0.05; ** *p* < 0.01; *** *p* < 0.001; **** *p* < 0.0001) compared to values from control mice. Data are representative of 5 independent experiments.

Splenocytes from scFv A1-treated or control MRL/lpr mice were additionally incubated in vitro with different concentrations of the scFv A1 antibody in order to study its ability to affect the cell proliferation. Significant dose-dependent stimulation of cell proliferation was observed in splenocytes from either young and sick scFv A1-treated mice, co-cultured with scFv A1 antibody compared to the cells from the untreated mice (Figure 4A). Stimulation of cell proliferation was also found in control cells from young scFv A1-treated animals cultured in medium only compared to untreated in vivo and in vitro mice. The same tendency was found when following the development of anti-dsDNA and anti-C1q antibody-secreting cells by an ELISpot assay used to assess how the scFv A1 antibody treatment affects the activity of plasma cells producing antibodies with these specificities. In vitro incubation of splenocytes from scFv A1 antibody-treated MRL/lpr mice and control animals with increasing amounts of scFv A1 antibody significantly increased the number of both anti-dsDNA and anti-C1q antibody-secreting plasma cells from in vivo scFv A1 antibody-treated animals compared to untreated controls in the groups of both young and old mice (Figure 4B). The same significant increase was monitored for anti-dsDNA antibody-secreting cells isolated from scFv A1 antibody-treated MRL/lpr mice without in vitro incubation compared to untreated mice in both young and old animals, while an insignificant increase was measured for anti-C1q antibody-secreting cells.

### 2.5. Weak Modulation of Proteinuria After scFv A1 Antibody Treatment

**Figure 5:**
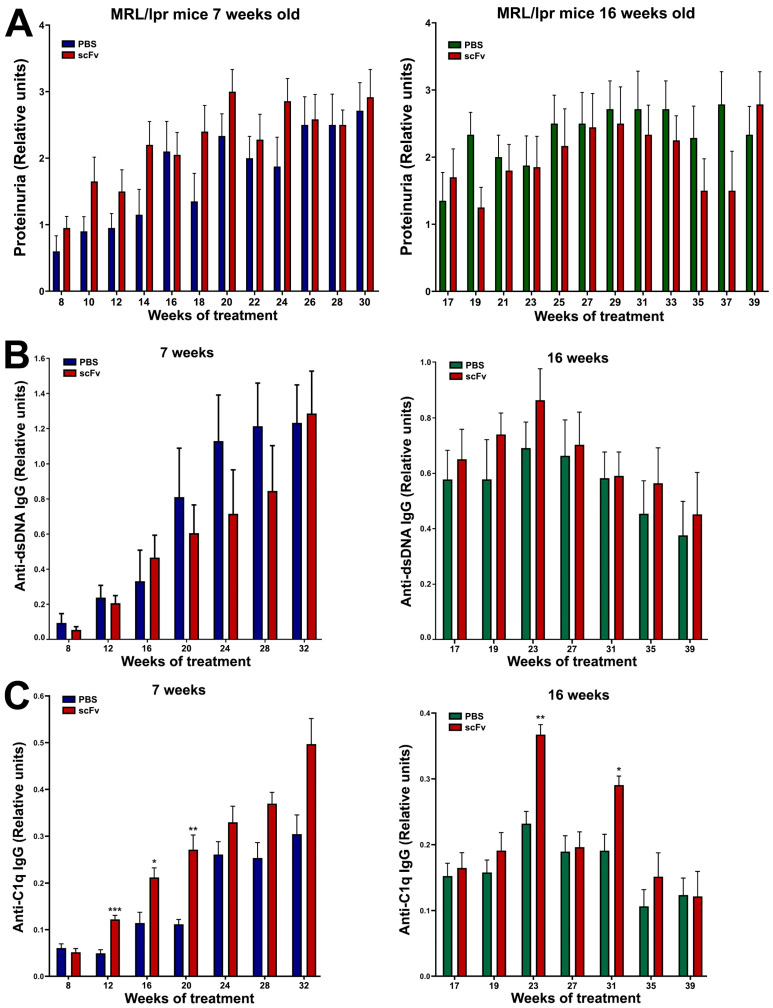
Administration of scFv A1 antibody to MRL/lpr mice resulted in the development of high levels of proteinuria in the young group of animals (**A**), increased values of IgG anti-dsDNA antibodies in the group of old mice (**B**), and high titers of IgG anti-C1q antibodies in both groups of animals (**C**) compared to untreated controls. All samples were triplicated and the average values were used for analysis. Mean ± SD values (*n* = 5) were calculated for each group; *p* values were calculated using a two-way ANOVA test (* *p* < 0.05; ** *p* < 0.01; *** *p* < 0.001).

A non-significant increase in urinary albumin was found after treatment of young MRL/lpr mice with scFv A1 antibody, while a non-significant decrease was measured after the same treatment of the group of old animals (Figure 5A).

### 2.6. ELISA for Anti-dsDNA and Anti-C1q IgG Antibodies

A huge increase in pathological anti-dsDNA IgG antibodies in MRL/lpr mice together with high levels of proteinuria are typical of lupus progression. Not surprisingly, high levels of anti-dsDNA IgG antibodies were found in both groups of animals in combination with high levels of proteinuria (Figure 5A,B). Treatment with the scFv A1 antibody suppressed non-significantly the rise of anti-dsDNA IgG antibodies in the group of young MRL/lpr mice, whereas treatment of old animals increased non-significantly the production of antibodies of this specificity. In contrast, scFv A1 antibody therapy further significantly increased anti-C1q IgG antibody levels in both young and old animals (Figure 5C).

### 2.7. Analysis of B- and T-Cell Apoptosis Ex Vivo

**Figure 6:**
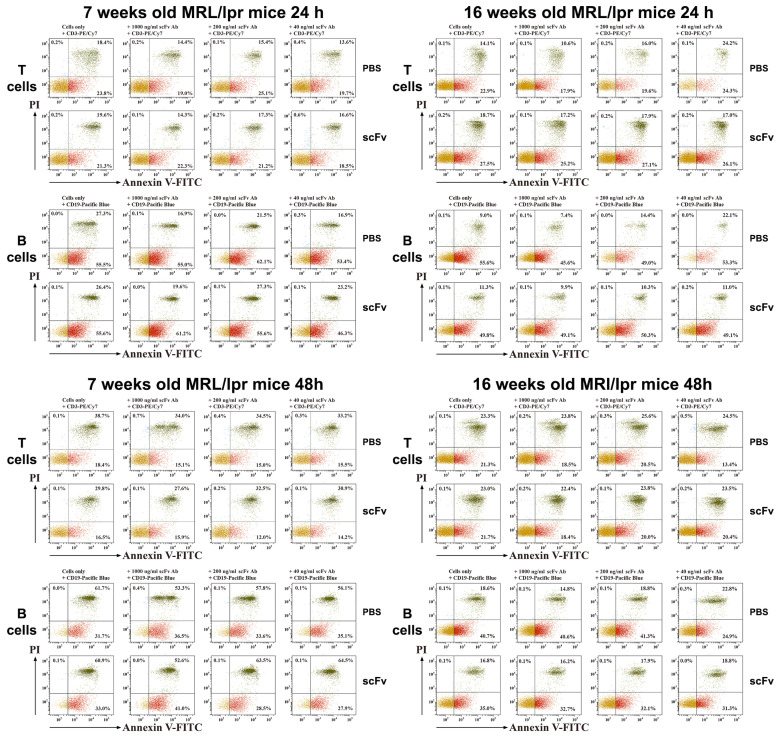
Ex vivo scFv A1 antibody treatment modulates T- and B-cell apoptosis. Spleen cells from all MRL/lpr mice were cultured in the presence of various concentrations of scFv A1 antibody as described in the text for Figure 4. Representative data from 5 experiments are shown.

T-lymphocytes isolated from scFv A1 antibody-treated MRL/lpr mice showed higher late apoptosis compared to controls after 24 h of incubation in both young and diseased animals. Additional ex vivo stimulation with scFv A1 antibody showed a dose-dependent change with the same trend (Figure 6). In contrast, after 48 h of ex vivo incubation with scFv A1 antibody, T-cells from 7- and 16-week-old mice administered in vivo with the same scFv showed lower levels of late apoptosis compared to mice treated with PBS. A greater variety of late cell apoptosis was found among B-lymphocytes. In groups of 7-week-old mice, 24 and 48 h of ex vivo incubation with scFv A1 antibody resulted in significantly higher levels of apoptosis in the group with in vivo scFv A1 antibody therapy compared to control animals. In contrast to young animals, late B-cell apoptosis in scFv A1 antibody-treated 16-week-old MRL/lpr mice showed generally lower values 24 and 48 h after ex vivo incubation with increasing amounts of the test molecule.

### 2.8. Lupus-Associated Cytokines Enhance Disease Progression

**Figure 7:**
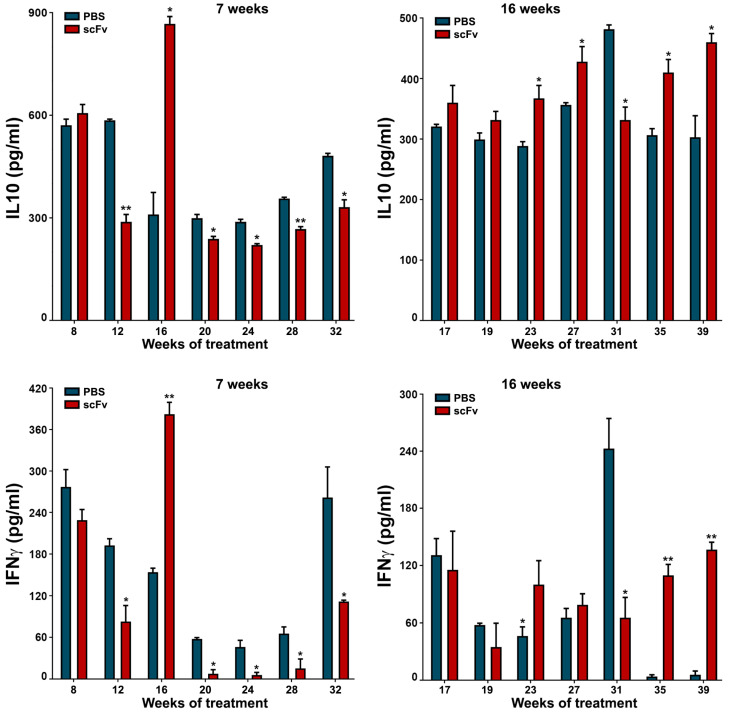
Therapy of lupus-prone MRL/lpr mice with scFv A1 antibody resulted in decreased values of IL10 and IFNγ when administered to young animals and increased levels of both cytokines after treatment of sick 16-week-old animals compared to untreated control mice. All samples were triplicated and average values were used for analysis. Mean ± SD values (*n* = 5) were calculated for each group; *p* values were calculated using a two-way ANOVA test (* *p* < 0.05; ** *p* < 0.01).

The disturbed balance between Th1/Th2 cytokines during the progression of lupus has a specific pattern. High values of IFNγ and IL10 in mouse sera are characteristic of the severe stage of the disease with unstable levels and fluctuations of these important for lupus cytokines (Figure 7). Regarding the young group of mice, scFv A1 antibody therapy significantly reduced the levels of both cytokines, excluding values at week 8 of treatment. Age discrimination was found in old MRL/lpr mice, and scFv A1 antibody treatment significantly increased IFNγ and IL10 values at most measurement points.

### 2.9. Kidney Histology

**Figure 8:**
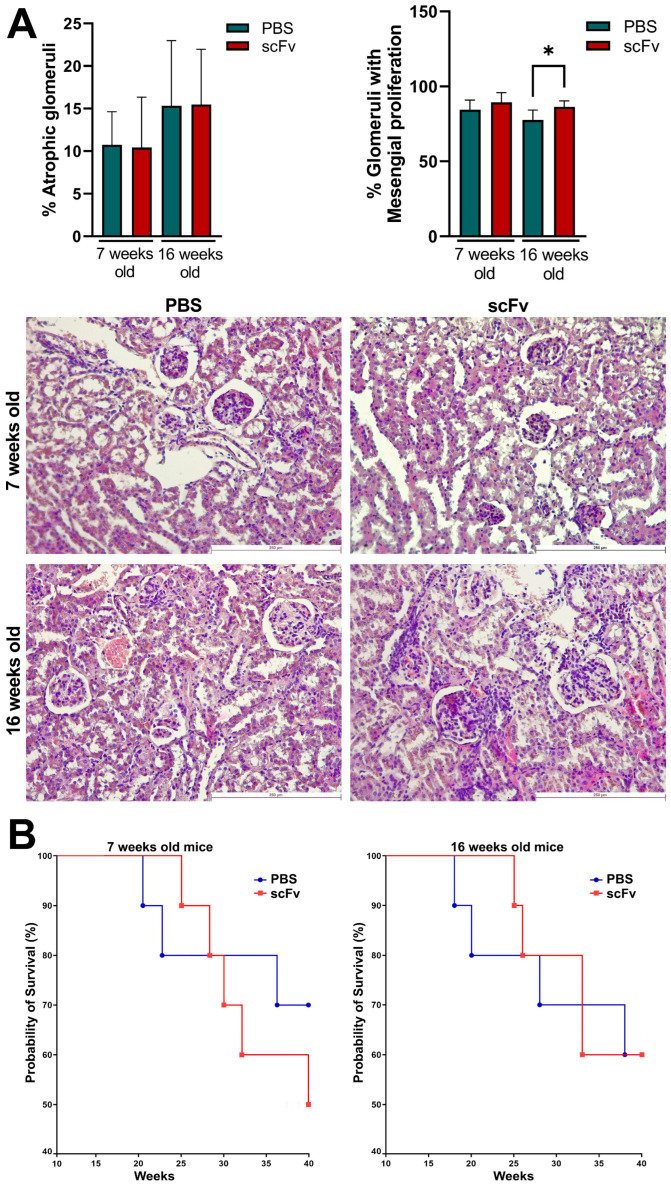
MRL/lpr mice treated with scFv A1 antibody exhibited pathological symptoms of lupus. (**A**) Histological kidney analyses: hematoxylin/eosin-stained kidney sections from young and old MRL/lpr mice. Sick 16-week-old animals treated with the engineered antibody showed an increased percentage of glomeruli with mesangial proliferation compared to mice untreated with scFv A1 antibody at the same age. Scale bar, 250 µm. Data are representative of 4–8 mice per group. Mean ± SD values were calculated for each group; *p* values were calculated using one-way Anova with post-test for multiple comparison (* *p* < 0.05); (**B**) Survival analyses in different experimental groups (*n* = 15 mice each) of 7-week-old (**left**) and 16-week-old (**right**) MRL/lpr mice after therapy with scFv A1 antibody. Survival significance was determined by analysis of survival curves using the Kaplan–Meier method. Representative data of 3 independent experiments are shown.

At the endpoint of the experiments, kidneys from all animals were removed for histological analysis. No significant differences were found in the percentage of atrophic glomeruli of scFv A1 antibody-treated mice compared to control animals in both young and old MRL/lpr mice (Figure 8A, left). The percentages of glomeruli with mesangial proliferation were significantly increased in 16-week-old mice after scFv A1 antibody therapy compared to mice treated with PBS alone. No significant differences were found between groups of 7-week-old mice (Figure 8A, right).

### 2.10. Survival Analysis

Treatment with the scFv A1 antibody significantly affected the survival of the young groups, and only 50% of the animals survived in the treated group at the end point of observation (week 30), compared to the untreated young MRL/lpr mice, which maintained a 70% survival rate (Figure 8B). No differences between the groups were found in the old animals with or without scFv A1 antibody therapy.

## 3. Discussion

SLE is a prototypic systemic autoimmune disease characterized by B- and T-cell hyperactivity and the presence of autoantibodies recognizing a variety of self-antigens. Most autoantibodies target intracellular structures or plasma proteins, and some of them are directly or indirectly involved in the pathogenesis of SLE. Among the pathogenic autoantibodies, anti-C1q antibodies play an important role in disrupting the proper mechanism of the complement system [18].

Along with its homeostatic functions, C1q is a well-documented autoantigen. This protein was first shown to be an autoantigen in SLE. Anti-C1q antibodies are predominantly IgG and are found in high titers in SLE patients with active lupus nephritis [24]. They bind with high affinity immobilized C1q, suggesting that they recognize neo-epitopes, exposed only at the conformational transition of C1q following its binding to a target surface. The first neo-epitopes were localized in CLR and the pathogenic role of anti-CLR in lupus nephritis kidneys was experimentally proven [25]. Later, autoepitopes in gC1q were also detected [26] by using recombinant forms of ghA, ghB, and ghC as test-antigens. It is yet to be understood why and how anti-gC1q antibodies appear, what is their clinical value, what is the relationship between the occurrence of anti-gC1q and anti-CLR and if any, and what factors affect it.

Many autoantibodies can be detected in patients with SLE several years before the onset of the disease. Among others, some autoantibodies are closely related to the severity of lupus and anti-C1q antibodies are considered to be involved in the pathogenesis of SLE and have the potential to accelerate the development of lupus [27]. Anti-C1q antibodies have been used as a diagnostic marker for the severity of lupus nephritis, but are also a factor in complement activation and deposition of immune complexes. Moreover, the physiological function of C1q to clear self-antigens generated during apoptosis is limited by the binding of anti-C1q antibodies in SLE patients [17].

Lupus-prone MRL/lpr mice are a suitable model to investigate potential therapeutic approaches for SLE based on specific antibody-targeted or cell-targeted treatment. Here, severe lupus disease is characterized by multiple organ involvement and overlap with human symptoms and pathologic findings. The MRL/lpr mouse model opens the framework to observe different autoantibodies binding dsDNA and other nuclear components, proteinuria, splenomegaly, renal failure and histological pathology, glomerulonephritis, skin lesions, and life span [21,22,23]. Cooperation between over-activated auto-reactive B-and T-lymphocytes in MRL/lpr mice promotes lupus progression resulting in formation of immune-complexes depositions and inflammatory cells infiltration [6,7].

The dynamics of the number of immune cells and their relative percentage among lymphocytes in the spleen and bone marrow are variable during the progression of lupus. Strong variations in cell numbers across cell types have been identified between disease-free and lupus MRL/lpr mice [28]. Administration of the scFv A1 antibody to both disease-free or sick MRL/lpr mice affected the amount of many important cell types and promoted inflammatory phenotype formation. A reduction in numbers of total B-cells has been previously described as a hallmark of the disease progression in MRL/lpr mice [29,30], but the scFv A1 antibody therapy additionally decrease significantly the total B-cell numbers in both young and old animals, providing evidence for severe lupus development. The increased numbers of Fo B-cells and Bregs observed in the spleens of both groups of mice after scFv A1 antibody treatment supported antibody production. In another study, the authors observed an increase in interaction between B-cells and CD4–/CD8+ conventional dendritic cells in the spleen of young MRL/lpr mice, suggesting an increase in B-cell activation [29]. Compared to untreated controls, a significant increase in the percentage of plasma cells and plasmablasts was found in the spleens of scFv A1 antibody-treated young and old animals, also suggesting stimulation of auto-antibody production.

While no significant changes of various activated T-cells were found, a significant decrease in the number of Tregs was detected in the old MRL/lpr group after scFv A1 antibody administration. In the spleens of sick MRL/lpr mice, a significant increase in interactions of CD4/CD8 double-negative B220+ T-cells (B220+ DN T-cells) with CD4+ T-lymphocytes, CD8+ T-lymphocytes, and other cell types was found compared to the number of these interactions in young MRL/lpr mice [29]. The close presence of B220+ DN T-cells next to CD4 T-cells in the spleens of MRL/lpr mice resulted in CD4 T-cell activation and increased levels of CD27 expression in CD4 T-cells. In this study, a non-significant increase in lupus-specific DN T-cells was measured in the old group of animals after scFv A1 antibody treatment, which may support disease progression.

NK-cells play diverse functions in murine lupus producing cytokines such as IFN-γ, TNF-α, GM-CSF, interleukin (IL)-10, and IL-13 upon stimulation. In the MRL/lpr mouse model, NK-cells migrated and infiltrated the kidneys of mice, which may be responsible for the renal damage seen in SLE [31]. No significant changes in NK-cell numbers were found among all subtypes in young 7-week-old animals after single-chain antibody therapy. In contrast, 16-week-old mice treated with scFv A1 antibody showed a dramatic rise in the levels of cytotoxic NK-cells (CD27−CD11b+) and a decrease in the number of early-maturing NK-cells (CD27+CD11b−) compared to controls. A decrease in the percentage of mature (CD27+CD11b+) NK-cells was also found in the same group of mice. All of these events can be responsible for enhanced lupus disease.

Hyperactivation of B- and T-cells is a typical feature of human and murine lupus. Nijnik et al. reported the presence of B-cell hyperactivity in MRL/lpr mice in the absence of self-antigen that contribute to the autoimmune phenotype of this mouse strain [32]. The authors associated this observation with a low total number of B-cells in the spleen and an expansion of the MZ B-cell population. In contrast, in this study, we observed a significant increase in the percentage of Fo B-cells after scFv A1 antibody therapy until disease severity increased. A significant increase in splenocyte proliferation was also measured in groups of in vivo treated mice after ex vivo scFv A1 antibody stimulation compared to untreated controls. Along with B-cell hyperactivation, the synergistic effect to increased overall splenocyte proliferation may cause activated DN T-cells and CD4 T-lymphocytes to form overall significantly increased proliferation rates [29].

A typical feature of SLE is low C1q levels and a possible reason is the presence of anti-C1q antibodies. The appearance of such antibodies in the serum of patients correlates with low complement activity and development of lupus nephritis. It is important to note that anti-C1q antibodies represent a wide spectrum of antibodies recognizing different C1q epitopes on the A-, B-, and C-chains of the polypeptide molecule [33]. In this study, in MRL/lpr mice, the presence of large numbers of plasma cells producing anti-C1q antibodies and high serum levels of antibodies of the same specificity was not surprising. Treatment of animals with scFv A1 antibody significantly increased both plasma cell counts and serum anti-C1q antibody levels, maintaining disease severity despite binding of scFv A1 to these autoantibodies. The possible reason is the binding of scFv A1 antibody to the BCR of the corresponding B-cells, which leads to the activation and proliferation of C1q-specific B-cells. With this therapy, we stimulate only one clone of anti-C1q-specific B-cells, but the possible mechanism of antigen spreading may expand the range of activated B-cell clones [34].

Interestingly, cross-reactivity of anti-DNA antibodies to an epitope in gC1q presented by the ghA was reported recently [35]. This may explain the decrease in anti-dsDNA antibodies in the 7-week-old mice after scFv A1 antibody treatment and the stable high levels of these autoantibodies in the 16-week-old animals after therapy.

Discussion of the role of C1q and anti-C1q antibodies in SLE has been driven by the “waste disposal” hypothesis based on the speculation that SLE is induced by a defective clearance of dying cells. This leads to the long-term presence of apoptotic cells in the circulation, which makes them antigenic and initiates autoimmunity. The C1q molecule is involved in the clearance of apoptotic cells in different ways. C1q can bind to apoptotic cells through interaction of its globular heads with phosphatidylserine external to the cell surface [18,36]. The collagen-like tails of C1q are then recognized by receptors on the surface of the phagocyte, which enhances the elimination of apoptotic cells. Furthermore, C1q rearranges the cytokine pattern of phagocytic cells during phagocytosis, which reduces the inflammatory response of macrophage-mediated inflammation, suggesting the multipotent function of C1q [18].

In our experiments, we can expect increased levels of C1q in the circulation caused by neutralization of anti-C1q antibodies by the administered scFv A1 antibody. This may lead to down-regulation of inflammation and suppression of lupus-related cytokines. Indeed, hyperactivated T-cells from scFv A1 antibody-treated animals showed increased late apoptosis after 24 h of ex vivo stimulation with the same engineered molecule. Surprisingly, a further 24 h of incubation reversed the apoptosis values and T-lymphocytes from untreated control mice showed higher levels of late apoptosis.

Moving to B-cells as a direct target of scFv A1 antibody injection, significantly higher levels of late apoptosis were measured in the group of 7-week-old mice with in vivo therapy with scFv A1 antibody, which marks the high presence of disease-associated B-cells during disease development. The lower values measured for B-cell apoptosis in 16-week- old animals confirmed the reduced B-cell numbers in mice with severe disease, when additional stimulation could no longer affect B-cells.

In a healthy immune system, IL10 and IFNγ play opposite roles in the frame of Th1/Th2 cytokine network. This is not the case in SLE: high levels of both cytokines can be found in the sera of lupus patients and lupus-prone mice [37]. IFNγ is produced by a wide range of lymphocytes, but during severe lupus, expanded B220+ DN T-cells are the major producers. IFNγ is responsible for Th1 stimulation and activation, strongly promoting lupus progression. Bregs and Tregs are producers of IL10, modulator of Th2 immune response. This regulatory cytokine suppresses the Th1 response but is a strong inducer of autoantibody production [38]. In this study, administration of scFv A1 antibody in 7-week-old mice resulted in significant suppression of both IL10 and IFNγ. In contrast, the therapy of sick 16-week-old animals enhanced lupus progression by significantly increasing both cytokines. During disease progression, the expansion of C1q-specific B-cell clones broadens the potential targets of the scFv A1 antibody and reorders cytokine production.

Although a direct link for the contribution of anti-C1q antibodies to the pathogenesis of lupus nephritis is still lacking, animal models of lupus provide data suggesting that renal inflammation is caused by preformed glomerular C1q-containing immune complexes induced by anti-C1q autoantibodies and requiring complement activation [18]. Lupus-prone MRL/lpr mice develop severe glomerulonephritis, and it is difficult to achieve even higher degrees of lupus nephritis with any treatment. High, erratic levels of proteinuria and hematuria are typical of this strain of mice over 12 weeks of age with strong individual characteristics of each animal. For this reason, only a minor increase in proteinuria was measured after therapy of 7-week-old animals with scFv A1 antibody with no differences between the 16-week-old groups. The same observations were found after histological analysis of stained kidneys from all groups of animals. As a logical reflection of the proteinuria, the renal damage of terminal control MRL/lpr mice reached a very high level of pathological score, and it is unrealistic to expect any difference in worsening of this pathology after scFv A1 antibody therapy. Such significant differences between the groups were found only for the score assessing glomeruli with mesangial proliferation in 16-week-old mice. As a logical consequence of the developed pathology, a difference in survival between groups was found only for 7-week-old mice with a worse survival rate for scFv A1 antibody-treated animals. No such difference was found for 16-week-old mice for the reasons mentioned above.

## 4. Materials and Methods

### 4.1. Antibodies

Anti-mouse Fluorescein isothiocyanate (FITC)-conjugated CD45, CD4, CD8, CD25, CD21/CD35, and CD335; eFlour450 or Pacific Blue-conjugated CD19 and CD3; Phycoerythrin (PE)-conjugated CD45, CD5, CD27, CD69, CD90.2, CD138, and FoxP3; Allophycocyanin (APC)-conjugated CD1d, CD4, CD11b, and CD25; Alexa Fluor (AF 700)-conjugated CD23 and PE-Cyanine 7 (PECy7)-conjugated CD8 mAbs (eBioscience, Frankfurt, Germany) were used for fluorescence-activated cell sorting (FACS) experiments. Relevant isotype-matched control IgG antibodies (eBioscience, Frankfurt, Germany) were used for compensation and primary antibody staining validation. Alkaline phosphatase (AP)-conjugated anti-mouse IgG antibody (Sigma-Aldrich, Taufkirchen, Germany) was used for enzyme-linked immunosorbent (ELISA) and enzyme-linked Immunospot (ELISpot) assays.

### 4.2. Expression and Purification of scFv A1

The expression and purification of scFv A1 was conducted according to Nikolova et al. [38]. Briefly, the synthesis of the recombinant antibody was induced in bacterial culture of *E. coli* HB 2151/A1 by a combined procedure which included alternating IPTG induction (0.5 mM IPTG (Fisher Bioreagents, Loughborough, UK) for 5 h at 25 °C) and autoinduction in ZYP-5052 medium (1% Tryptone (Casitose Type-I, HiMedia, Mumbai, India), 0.5% Yeast Extract (HiMedia, Mumbai, India), 25 mM (NH_4_)_2_SO_4_ (Chem-Lab NV, Zedelgem, Belgium), 50 mM KH_2_PO_4_ (Fisher Bioreagents, Loughborough, UK), 50mM Na_2_HPO_4_ (Fisher Bioreagents, Loughborough, UK), 0.5% glycerol (Sigma, St. Louis, MO, USA), 0.05% glucose (Merck KGaA, Gernsheim, Germany), 0.2% α-lactose (Carl Roth GmbH, Karlsruhe, Germany), 1 mM MgSO_4_ (Fisher Scientific, Loughborough, UK)), containing 100 g/mL Ampicillin (Fisher Bioreagents, Pittsburg, PA, USA) for 16 h at 25 °C. The induced cells were harvested by centrifugation and subjected to a two-step lysis with buffer 1 (100 mM Tris-HCl, pH.8.0, 20% sucrose (Fisher Scientific, Loughborough, UK) and 1 mM EDTA (Fisher Bioreagents, Fair Lawn, NJ, USA) for 1 h on ice and then with buffer 2 (5 mM MgSO_4_) for 15 min on ice. The resulting supernatants were dialysed for 24 h at 4 °C against phosphate buffer, pH 8.0 containing 10 mM imidazole. After dialysis, the supernatants were pooled and filtered through 0.45 μm filter, and then loaded on HIS-Select^®^ Ni-affinity gel (Acros Organics, Fair Lawn, NJ, USA) column, pre-equilibrated in phosphate buffer, pH 8.0 containing 10 mM imidazole and 0.02% NaN_3_, at a flow rate of 0.5 mL/min. The His-tagged scFv antibodies were eluted with phosphate buffer, pH 8.0 containing 250 mM imidazole and 0.02% NaN_3_. The eluted protein samples were dialysed for 24 h at 4 °C against PBS, pH 7.2. The protein concentration was determined by measuring the optical density at 280 nm (OD280).

### 4.3. Mice

Female MRL/lpr (MRL/MpJ-Tnfrsf6lpr/J) mice were obtained from The Jackson Laboratory, (Bar Harbor, ME, USA) and maintained in our barrier-type animal facility under specific-pathogen-free (SPF) conditions. Groups of 7-week-old female mice with initial disease and 16-week-old mice with severe disease were used for our experiments.

The animals were randomly assigned to the respective groups (five animals per cage) and the manipulations were approved by the Animal Care Commission at the Institute of Microbiology in accordance with International regulations (EU Directive 2010/63/EU).

### 4.4. Treatment Schedule

Groups of 7-week-old and 16-week-old MRL/lpr mice (10 animals each) were injected every 7 days intraperitoneally (i.p.) with 20 µg/mouse of scFv A1 antibody in 100 µL PBS. Treatment was administered for five weeks to perform FACS analyses and up to 39 weeks to monitor autoantibody and cytokine levels during lupus progression. Two control age-matched animal groups (10 animals each) were treated with the same volume of vehicle (PBS) only. Every two weeks the animals were bled and the sera were kept frozen at −80 °C for further analyses.

### 4.5. Flow Cytometry

Spleens were taken from sacrificed MRL/lpr mice after five weeks of treatment with scFv A1 antibody and monocellular suspensions prepared as described [23]. Bone marrow cells were isolated from mouse tibia. The muscles and residue tissues surrounding the femur were removed with sterile forceps and scissors. Later, a 23-gauge needle and a syringe filled with RPMI (Roswell Park Memorial Institute) 1640 medium (GE Healthcare, Hatfield, UK) were used to flush the bone marrow onto a 70 μm cell strainer (BD Biosciences, Erenbodegem, Belgium) placed into a sterile cell culture dish. The bone marrow was smashed through the cell strainer with a plunger and washed with RPMI. The cell suspension was centrifuged at 1200 rpm for 10 min at 4 °C, the pellet was homogenized with 1 mL RBC lysis buffer and incubated for 5 min at RT. The cell solution was neutralized by adding RPMI, the cells were washed by centrifugation and suspended in RPMI medium supplemented with 10% heat-inactivated fetal calf serum (FCS from Capricorn Scientific, Ebsdorfergrund, Germany), 1 mM sodium pyruvate, 4 mM L-glutamine, and antibiotics (all from Sigma). The number of splenocytes and bone marrow cells was counted by a haemocytometer.

One of the following mixes of anti-mouse antibodies were used for incubation: FITC-conjugated CD45R and Pacific Blue-conjugated CD19 antibodies for B-cell populations; PE-conjugated CD45R, Pacific Blue-conjugated CD19, APC-conjugated CD1d, FITC-conjugated CD21/CD35, and AlexaFluor700-conjugated CD23 antibodies for differentiation of marginal zone (MZ) and Follicular (Fo) B-cells; FITC-conjugated CD45R, Pacific Blue-conjugated CD19, and PE-conjugated CD138 antibodies for plasma cells; eFluor450-conjugated CD19, PE-conjugated CD5, and APC-conjugated CD1d antibodies for regulatory B (Breg) cells; PE-conjugated CD45R, eFluor450-conjugated CD3, APC-conjugated CD4, and FITC-conjugated CD8 antibodies for CD4 and CD8 T-cell populations; Pacific Blue-conjugated CD3, APC-conjugated CD4, FITC-conjugated CD8, and PE-conjugated CD90.2 antibodies for CD4/CD8 double-negative activated T-cells; Pacific Blue-conjugated CD3, APC-conjugated CD4, PECy7-conjugated CD8, FITC-conjugated CD25 and PE-conjugated CD69 antibodies for activated CD4 and CD8 T-cells; Pacific Blue-conjugated CD3, FITC-conjugated CD4, PE-conjugated FoxP3, and APC-conjugated CD25 antibodies for regulatory T-cells (Tregs); eFlour 450-conjugated CD3e, FITC-conjugated CD335, APC-conjugated CD11b, and PE-conjugated CD27 antibodies for NK population and activated NK-cells. The cells were incubated with respective combinations of fluorochrom-conjugated antibodies for staining of B-, T-, and NK-cell subpopulations (1 µg/10^6^ cells) for 20 min on ice. Thirty thousand cells were analyzed from each sample with a BD LSR II flow cytometer (BD Biosciences, San Jose, CA, USA). Intact cells were gated using forward and side scatter and the data was analyzed with the Diva 6.1.1. software.

### 4.6. Proliferation Assay

The MTT (3-[4,5-dimethylthiazol-2-yl]-2,5-diphenyltetrazolium bromide; thiazolyl blue) assay was performed as described [23]. Briefly, the isolated splenocytes (2 × 10^6^/mL) from control and scFv A1-treated groups of MRL/lpr mice were co-cultured for 3 days in RPMI 1640 medium containing 10% FCS, 4 mM L-glutamine, and antibiotics in the presence of different concentrations of scFv A1 antibody (ranging from 8 ng/mL to 1000 ng/mL) at 37 °C/5% CO_2_. Control cells were stimulated either with lipopolysaccharide (LPS, 10 μg/mL) (from Sigma-Aldrich), concanavalin A (ConA, 10 µg/mL) (from Sigma-Aldrich), or cultured in RPMI medium only. Later, the splenocytes were proceeded under standard procedures for MTT or ELISpot development.

### 4.7. ELISpot Assay for Counting Specific Anti-dsDNA and Anti-C1q Antibody-Secreting Cells

The level of generated IgG anti-dsDNA and anti-C1q antibody-secreting cells was assessed by ELISpot assay. The isolated splenocytes from scFv A1 antibody-treated mice and control animals were cultured as described above. Next, the ELISpot assay was performed as previously described [39]. Briefly, ethanol-activated 96-well ELISpot plates were coated with calf thymus dsDNA (10 µg/mL, from Sigma) or C1q (5 µg/mL, Merck, Darmstadt, Germany). scFv A1 pre-incubated splenocytes were transferred from the culture plates into the DNA- or C1q-coated ELISpot plates and were additionally cultured for 4 h. The plates were then incubated with an AP-conjugated anti-mouse IgG and developed. The counted spots correspond to the number of plasma cells producing IgG anti-dsDNA or anti-C1q antibodies (C.T.L Immunospot S5 Versa Analyzer, Bonn, Germany).

### 4.8. Proteinuria Measurement

The levels of protein in urine were measured weekly directly from the urethra using Combiscreen 11 SYS PLUS strips (Analyticon Biotechnologies, Lichenfels, Germany) and scored semi-quantitatively as: (0—none; 1—30 to 100; 2—100 to 300; 3—300 to 500, and 4—>500 mg dL^−1^).

### 4.9. ELISA for Anti-dsDNA and Anti-C1q IgG Antibodies

The IgG anti-dsDNA antibodies were measured in mouse sera by ELISA as previously described [22]. The IgG anti-C1q antibody levels were detected by coating of immunoassay plates with 1 µg/mL C1q (Merck Millipore Calbiochem™, Darmstadt, Germany) diluted in coating buffer (0.2 M NaHCO_3_, pH 9.6) for 16 h at 4 °C. The plates were extensively washed with 0.05% Tween in PBS (T-PBS) and blocked with 1% bovine serum albumin (BSA, Sigma) for 1 h at 37 °C. Then the plates were incubated with serum samples and controls diluted 50× in dilution buffer (0.05% Tween, 1% FBS, 1 M NaCl in PBS) for 2 h at 37 °C. The plates were washed and AP-conjugated anti-mouse IgG antibody (3000× diluted) was added for 1 h at 37 °C. The reaction was developed with p-nitrophenylphosphate (pNPP) substrate (Sigma-Aldrich) and read at 405 nm. A mouse anti-dsDNA IgG2a antibody 10F10 (kindly provided by Dr Bor-Luen Chiang, College of Medicine, National Taiwan University, Taipei, Taiwan) was used as standard for anti-dsDNA IgG antibody determination.

### 4.10. Apoptosis Assay

Apoptosis values of gated B- or T-cells were assessed as described [23]. Briefly, splenocytes from scFv A1 antibody–treated MRL/lpr mice or untreated PBS control animals (2 × 10^6^ cells/mL) were cultured in supplemented RPMI 1640 medium for 24 or 48 h at 37 °C/5% CO_2_ in the presence of increasing concentrations of scFv A1 antibody (ranging from 40 ng/mL to 1000 ng/mL). Later, the splenocytes were washed and stained with Pacific Blue-conjugated anti-mouse CD19 or PE/Cy7-conjugated anti-mouse CD3 antibodies. The apoptosis of B- and T-cells was assessed by FACS using the Annexin V-FITC apoptosis detection Kit (eBioscience, Frankfurt, Germany).

### 4.11. Cytokine Detection

IFNγ and IL10 levels were measured in mouse sera from all animals using ELISA MAXTM Deluxe Set (BioLegend, Waltham, MA, USA) according to the manufacturer’s instructions.

### 4.12. Kidney Histology

The protocols were performed as previously described [22]. Briefly, kidneys from all mice were removed and fixed in formalin, embedded in paraffin, and then sectioned. Paraffin sections were stained with hematoxylin/eosin using a standard staining technique for histopathological examination. Kidney sections were examined and imaged using a Leica DM2000 microscope (Leica Biosystems, Wetzlar, Germany). The presence of glomerular atrophy and mesangial glomerular proliferation (>3 cells per mesangial area) were estimated and the results are shown as percentages of atrophic glomeruli or of glomeruli with mesangial proliferation. Mesangial proliferation was determined as mild—affecting up to 25% from the kidney, moderate—affecting up to 50% from the kidney tissue, and severe—affecting >50% from the kidney tissue. The observations were conducted by an investigator who was blinded to the experimental groups.

### 4.13. Statistical Analysis

All statistical analyses were performed with Prism software v.9 from GraphPad (San Diego, CA, USA). The one-way and two-way ANOVA tests were used to determine differences between each two groups. All ELISA, proliferation, and cytokine samples were triplicated. Survival significance was determined using the method of Kaplan–Meier. Values in the figures correspond to mean ± SD and *p* < 0.05 was considered as statistically significant.

## 5. Conclusions

The anti-idiotypic scFv A1 antibody exhibits dual properties in terms of its function and target recognition in the immune system. After injection, this engineered molecule can bind circulating anti-C1q antibodies, which suppresses autoimmune development and the formation of potential immune complexes. In contrast, treatment with the same scFv A1 antibody resulted in BCR recognition on the surface of anti-C1q-specific B-cells and had a disease progression effect, enhancing lupus symptoms in the MRL/lpr mouse model of SLE. These biological risks are a challenge for all newly developed molecules for biological therapy of lupus and must be tested in preclinical animal trials.

## Data Availability

The data presented in this study are available on request from the corresponding author.

## Abbreviations

The following abbreviations are used in this manuscript:

SLE	Systemic lupus erythematosus
scFv	Single-chain variable fragment
gC1q	Globular domains
CLR	Collagen-like (CLR) domain
Fo B	Follicular B-cells
MZ B	Marginal zone B lymphocytes
Bregs	Regulatory B-cells
Tregs	Regulatory T-cells
RPMI	Roswell Park Memorial Institute Medium
LPS	Lipopolysaccharide
ConA	Concanavalin A

## References

[1] Rojas M., Rodríguez Y., Joan Leon K., Pacheco Y., Acosta-Ampudia Y., Monsalve D.M., Ramírez-Santana C., Anaya J.M. (2018). Cytokines and Inflammatory Mediators in Systemic Lupus Erythematosus. EMJ Rheumatol..

[2] Ferretti C., La Cava A., Tsokos G.C. (2016). Chapter 8—Overview of the Pathogenesis of Systemic Lupus Erythematosus. Systemic Lupus Erythematosus.

[3] Zeng J., Wu H., Zhao M., Lu Q. (2017). Novel biomarkers for systemic lupus erythematosus. Biomark. Med..

[4] Dossybayeva K., Abdukhakimova D., Poddighe D. (2020). Basophils and Systemic Lupus Erythematosus in Murine Models and Human Patients. Biology.

[5] Shah K., Cragg M., Leandro M., Reddy V. (2021). Anti-CD20 monoclonal antibodies in Systemic Lupus Erythematosus. Biol. J. Int. Assoc. Biol. Stand..

[6] Richard M.L., Gilkeson G. (2018). Mouse models of lupus: What they tell us and what they don’t. Lupus Sci. Med..

[7] Rottman J.B., Willis C.R. (2010). Mouse models of systemic lupus erythematosus reveal a complex pathogenesis. Vet. Pathol..

[8] Dörner T., Giesecke C. (2011). Lipsky PE Mechanisms of B cell autoimmunity in SLE. Arthritis Res. Ther..

[9] Foster M.H. (2007). T cells and B cells in lupus nephritis. Semin. Nephrol..

[10] Tai Y., Wang Q., Korner H., Zhang L., Wei W. (2018). Molecular mechanisms of T cells activation by dendritic cells in autoimmune diseases. Front. Pharmacol..

[11] Wilkinson M.G.L., Rosser E.C. (2019). B cells as a therapeutic target in paediatric rheumatic disease. Front. Immunol..

[12] Ahuja A., Shupe J., Dunn R., Kashgarian M., Kehry M.R., Shlomchik M.J. (2007). Depletion of B cells in murine lupus: Efficacy and resistance. J. Immunol..

[13] Kansal R., Richardson N., Neeli I., Khawaja S., Chamberlain D., Ghani M., Ghani Q.-U.-A., Balazs L., Beranova-Giorgianni S., Giorgianni F. (2009). Sustained B cell depletion by CD19-targeted CAR T cells is a highly effective treatment for murine lupus. Sci. Transl. Med..

[14] Rovin B.H., Furie R., Latinis K., Looney R.J., Fervenza F.C., Sanchez-Guerrero J., Maciuca R., Zhang D., Garg J.P., Brunetta P. (2012). Efficacy and safety of rituximab in patients with active proliferative lupus nephritis: The Lupus Nephritis Assessment with Rituximab study. Arthritis Rheum..

[15] Wallace D.J., Kalunian K., Petri M.A., Strand V., Houssiau F.A., Pike M., Kilgallen B., Bongardt S., Barry A., Kelley L. (2014). Efficacy and safety of epratuzumab in patients with moderate/severe active systemic lupus erythematosus: Results from EMBLEM, a phase IIb, randomised, double-blind, placebo-controlled, multicentre study. Ann. Rheum. Dis..

[16] Stojan G., Petri M. (2016). Anti-C1q in systemic lupus erythematosus. Lupus.

[17] Csorba K., Schirmbeck L.A., Tuncer E., Ribi C., Roux-Lombard P., Chizzolini C., Huynh-Do U., Vanhecke D., Trendelenburg M. (2019). Anti-C1q Antibodies as Occurring in Systemic Lupus Erythematosus Could Be Induced by an Epstein-Barr Virus-Derived Antigenic Site. Front. Immunol..

[18] Thanei S., Trendelenburg M. (2016). Anti-C1q Autoantibodies from Systemic Lupus Erythematosus Patients Induce a Proinflammatory Phenotype in Macrophages. J. Immunol..

[19] Kishore U., Gupta S.K., Perdikoulis M.V., Kojouharova M.S., Urban B.C., Reid K. (2003). Modular Organization of the Carboxyl-Terminal, Globular Head Region of Human C1q A, B, and C Chains. J. Immunol..

[20] Todorova N., Rangelov M., Bogoeva V., Stoyanova V., Yordanova A., Nikolova G., Georgiev H., Dimitrova D., Mohedin S., Stoyanova K. (2021). Anti-Idiotype scFv Localizes an Autoepitope in the Globular Domain of C1q. Int. J. Mol. Sci..

[21] Tchorbanov A.I., Voynova E.N., Mihaylova N.M., Todorov T.A., Nikolova M., Yomtova V.M., Chiang B., Vassilev T.L. (2007). Selective silencing of DNA-specific B lymphocytes delays lupus activity in MRL/lpr mice. Eur. J. Immunol..

[22] Mihaylova N., Bradyanova S., Chipinski P., Chausheva S., Kyurkchiev D., Tchorbanov A. (2020). Monoclonal antibody therapy that targets phospholipid-binding protein delays lupus activity in MRL/lpr mice. Scand. J. Immunol..

[23] Bradyanova S., Mihaylova N., Chipinski P., Manassiev Y., Herbáth M., Kyurkchiev D., Prechl J., Tchorbanov A. (2021). Anti-ANX A1 Antibody Therapy in MRL/lpr Murine Model of Systemic Lupus Erythematosus. Arch. Immunol. Ther. Exp..

[24] Trendelenburg M. (2005). Antibodies against C1q in patients with systemic lupus erythematosus. Springer Semin. Immunopathol..

[25] Trouw L.A., Groeneveld T.W.L., Seelen M.A., Duijs J.M.G.J., Bajema I.M., Prins F.A., Kishore U., Salant D.J., Verbeek J.S., van Kooten C. (2004). Anti-C1q autoantibodies deposit in glomeruli but are only pathogenic in combination with glomerular C1q-containing immune complexes. J. Clin. Investig..

[26] Tsacheva I., Radanova M., Todorova N., Argirova T., Kishore U. (2007). Detection of autoantibodies against the globular domain of human C1q in the sera of systemic lupus erythematosus patients. Mol. Immunol..

[27] Beurskens F.J., van Schaarenburg R.A., Trouw L.A. (2015). C1q, antibodies and anti-C1q autoantibodies. Mol. Immunol..

[28] Goltsev Y., Samusik N., Kennedy-Darling J., Bhate S., Hale M., Vazquez G., Black S., Nolan G.P. (2018). Deep Profiling of Mouse Splenic Architecture with CODEX Multiplexed Imaging. Cell.

[29] Shlomchik M.J., Madaio M.P., Ni D., Trounstein M., Huszar D. (1994). The role of B cells in lpr/lpr-induced autoimmunity. J. Exp. Med..

[30] Liu M., Liang S., Zhang C. (2021). NK Cells in Autoimmune Diseases: Protective or Pathogenic?. Front. Immunol..

[31] Nijnik A., Ferry H., Lewis G., Rapsomaniki E., Leung J.C., Daser A., Lambe T., Goodnow C.C., Cornall R.J. (2006). Spontaneous B cell hyperactivity in autoimmune-prone MRL mice. Int. Immunol..

[32] Kleer J.S., Rabatscher P.A., Weiss J., Leonardi J., Vogt S.B., Kieninger-Gräfitsch A., Chizzolini C., Huynh-Do U., Ribi C., Trendelenburg M. (2022). Epitope-Specific Anti-C1q Autoantibodies in Systemic Lupus Erythematosus. Front. Immunol..

[33] Fahlquist-Hagert C., Wittenborn T.R., Terczyńska-Dyla E., Kastberg K.S., Yang E., Rallistan A.N., Markett Q.R., Winther G., Fonager S., Voss L.F. (2023). Antigen presentation by B cells enables epitope spreading across an MHC barrier. Nat. Commun..

[34] Franchin G., Son M., Kim S.J., Ben-Zvi I., Zhang J., Diamond B. (2013). Anti-DNA antibodies cross-react with C1q. J. Autoimmun..

[35] Kapogianni A., Radulova G., Donev V., Videv P., Cholakova G., Iliev S., Ivanova A., Bogoeva V., Tsacheva I. (2025). Characterization of the binding of the globular domains of the complement component C1q to phosphatidylserine. Int. J. Biol. Macromol..

[36] Blenman K., Duan B., Xu Z., Wan S., A Atkinson M., Flotte T.R., Croker B.P., Morel L. (2006). IL-10 regulation of lupus in the NZM2410 murine model. Lab. Investig..

[37] Rasquinha M.T., Sur M., Lasrado N., Reddy J. (2021). IL-10 as a Th2 Cytokine: Differences Between Mice and Humans. J. Immunol..

[38] Nikolova G., Georgieva Y., Atanasova A., Radulova G., Kapogianni A., Tsacheva I. (2021). Autoinduction as Means for Optimization of the Heterologous Expression of Recombinant Single-Chain Fv (scFv) Antibodies. Mol. Biotechnol..

[39] Mihaylova N., Bradyanova S., Chipinski P., Herbáth M., Chausheva S., Kyurkchiev D., Prechl J., Tchorbanov A.I. (2017). Annexin A1 as a target for managing murine pristane-induced systemic lupus erythematosus. Autoimmunity.

